# Metformin prevents murine ovarian aging

**DOI:** 10.18632/aging.102016

**Published:** 2019-06-10

**Authors:** Xian Qin, Dingfu Du, Qian Chen, Meng Wu, Tong Wu, Jingyi Wen, Yan Jin, Jinjin Zhang, Shixuan Wang

**Affiliations:** 1 Department of Obstetrics and Gynecology, Tongji Hospital, Tongji Medical College, Huazhong University of Science and Technology, Wuhan, Hubei, People’s Republic of China; 2 Department of Obstetrics and Gynecology, The First Affiliated Hospital of Chongqing Medical University, Chongqing, People’s Republic of China

**Keywords:** metformin, ovarian aging, SIRT1, oxidative stress

## Abstract

A number of studies have shown that metformin can delay aging process and extend healthy lifespan in animals. However, its role in female reproductive lifespan is unclear. This study was aimed to explore the potential anti-aging effect of metformin on the ovary and its possible mechanisms. Female C57BL/6 mice of 27-week old were divided into two groups, the control group (CON) and metformin-treated group (MET). CON mice were fed ad libitum, while MET mice were fed on chows supplied with 100mg/kg metformin for half a year. Ovarian reserve and function were assessed by ovarian follicle counts, estrous cycle and sex hormones levels. The expressions of oxidized metabolites, such as 8-hydroxy-2´-deoxyguanosine (8-OHdG), 4-hydroxynonenal (4-HNE), nitrotyrosine (NTY), and ovarian aging associated proteins P16, SIRT1, p-rpS6 and Bcl2 were examined. The MET mice exhibited increased level of serum E2 hormone and higher percentage of regular estrous cycles after 6 months' feeding, compared to the CON mice. The amount of primordial and primary follicles and the expression of SIRT1 were significantly increased, but the levels of P16, 8-OHdG, 4-HNE and p-rpS6 were decreased in the MET mice. These results indicate that metformin can delay ovarian aging process, probably by inducing the expression of SIRT1 and reducing the oxidative damage.

## INTRODUCTION

The ovary, regarded as an aging pacemaker, appears to age at the earliest stage during a woman’s lifespan [1]. Ovarian aging refers to the decline of female ovarian functions with age, accompanied by the deterioration in the quantity and quality of ovarian follicles, and ends with menopause. This aging process is complicated and affected by a number of factors, including lifestyle, medical, genetic, autoimmune, environmental, and idiopathic ones. It is also often highly associated with other disorders in women. Over the past decade, with the delay of women's child-bearing age, ovarian aging-induced reproductive problems have become a big issue to the mankind. Also, as the average lifespan extends, an increasing number of women will spend nearly one-third of their lives in a post-menopausal state [2]. Menopause is the final step of ovarian aging and leads to lots of health disorders, such as osteoporosis, cardiovascular disease, tumor, Alzheimer's disease, obesity, diabetes and so on [3]. Thus, it is crucially important to postpone ovarian aging and improve ovarian functions. Although a marked progress has been made in the exploration of anti-ovarian aging agents or approaches, such as calorie restriction mimetics, antioxidants, autophagy inducers, epigenetic drugs, immunomodulator etc., over the past years, none of these have become clinically effective or useful.

One of the extensively studied anti-aging agents is metformin. Metformin is a prescribed drug approved to treat diabetes. Beyond the antihyperglycemic effects, metformin can exert multiple anti-aging actions at the cellular and organismal levels [4]. Currently, a number of studies have indicated that metformin can delay aging process and extend healthy lifespan in various animal model systems, such as worms [5], drosophila [6, 7], and rodents [8–11]. Metformin leads to the decrease of insulin levels and IGF-1 signaling [12], inhibition of the mTOR pathway and mitochondrial complex 1 in the electron transport chain [13], activation of AMP-activated kinase (AMPK) [6], reduction of DNA damage [7], and endogenous production of reactive oxygen species (ROS) [11]. Metformin favorably influences metabolic and cellular processes, which are closely associated with the development of age-related conditions, such as inflammation [14], autophagy [15], and cellular senescence [16]. Recent studies indicated that metformin extends lifespan by altering the microbiome, specifically by changing microbial folate and methionine metabolism [17]. These findings prompted us to explore the possibility of that metformin might also be able to delay the ovarian aging process. In our attempt to address this question by employing mice, we found that indeed metformin treatment for half a year can delay ovarian aging process and improve ovarian functions as assessed by analyzing several molecular markers and ovarian functions.

## RESULTS

### Long-term treatment of mice with metformin is relatively safe

The aging of female ovary is accompanied by the depleted follicles numbers and diminished quality of oocytes. The female ovarian function appears to decline sharply at the age of 37 years old [18]. Previous studies have indicated that the corresponding age in mice is about 28 weeks [19]. Also, one study suggested that the health lifespan of C57/BL mice could be prolonged by feeding with 100mg/kg metformin [11]. Thus, mice of 28-week old were selected in this study, then 100mg/kg metformin was given for half a year to explore whether the intervention of recommended metformin dosage could delay the ovarian aging process and extend reproductive span.

**Figure 1:**
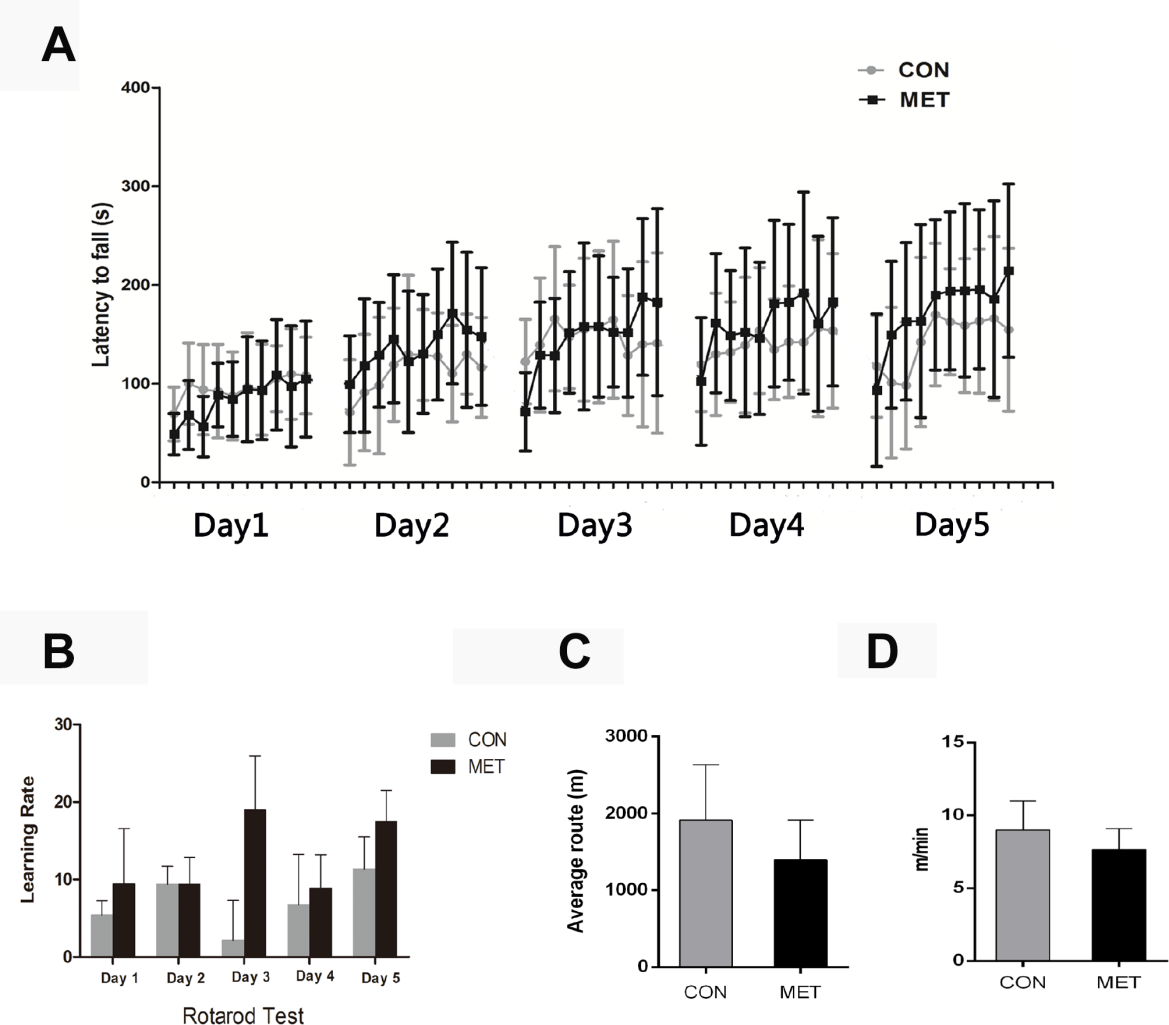
**Behavior tests of CON and MET mice after 6-months’ feeding.** (**A**), Rotarod Test; (**B**), Learning Rate of mice trained on the accelerating rotarod; (**C**), Open-field Test; (**D**), Average route of mice in the open-field. Data were represented as mean± S.E.M.

Before we tested if metformin might affect ovarian aging, we first determined whether the long-term treatment of mice with a moderate dosage (100 mg/kg) of metformin is safe to mice or not. After this treatment for 6 months, some mice died of natural causes. We found that both of the CON and MET alive mice are quite healthy, and their body weights and organ weights showed no significant difference between the two groups (Table 1). To test the physical activities and whole body state of the mice, we conducted the average speed, average routine and latency to fall of the animals in the open-field test and in rotarod test, respectively, and found that there is no significant difference between the two groups, either. Interestingly, the MET mice tend to show a higher learning rate compared with the CON mice but with no significant difference, which indicate that MET may improve the memory of mice (Figure 1). Thus, these results indicate that the dosage of metformin used in this study is safe to the animals and thus will be used in the following experiments.

**Table 1:** The effect of metformin on organ index of mice after 6-month feeding (data is presented as mean ± S.E.M).

**Group**	**Body Weight(g)**	**Ovarian weight(mg)**	**Ovary (mg/g)**	**Heart (mg/g)**	**Liver (mg/g)**	**Spleen (mg/g)**	**Kidney (mg/g)**	**Brain (mg/g)**	**Uterus (mg/g)**
CON	26.58±1.92	2.38±0.77	0.11±0.02	6.86±1.07	52.98±8.68	6.42±2.25	7.18±0.59	18.31±1.38	3.31±1.09
MET	25.88±2.93	2.92±0.58	0.09±0.02	6.44±0.99	53.00±3.75	6.55±1.73	7.16±0.60	18.96±1.54	3.56±0.76

### Metformin improves ovarian function

Next, we assessed the effect of metformin on mouse ovarian functions by first examining estrous cycles and hormones levels, as they can reflect the ovarian endocrine function. In this study, the 4-5 day estrous cycle was judged as a normal estrous cycle, and the estrous cycle lasting longer than 5 days or less than 4 days was considered as an unnormal estrous cycle. Only mice with normal estrous cycle for at least 4 times continuously in a month was regarded as with regular estrous cycle. Mice with the sustained estrous period or the intermittent normal estrous cycle was regarded as irregular. The criteria for the cessation of estrous cycle was the absence or little of cells in the vaginal smear for at least 20 days [20, 21].

**Figure 2:**
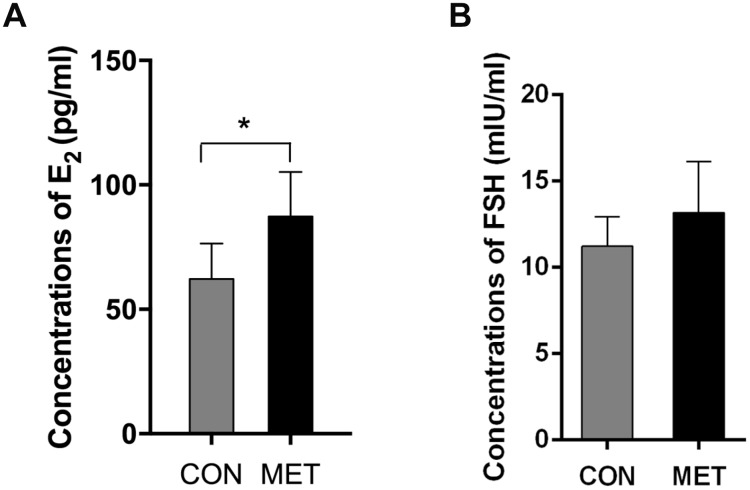
**E2 and FSH levels in both groups.** Data were represented as mean± S.E.M. *Compared with the CON group, *P<0.05

As shown in Table 2, the estrous cycles of mice in both groups showed no significant difference after 3-month feeding, with the ratio of regular cycles of 85.7% in CON mice over 85% in MET mice. However, after feeding the animals with metformin-containing foods for 6 months, the regular cycle ratio of the CON mouse ovaries dropped to 38.5%, while that in the MET mice still maintained quite high at 78.9%. This result indicates that metformin protects the murine ovaries from aging by maintaining its ovarian regular cycle at a higher level. Consistent with this result, the level of E2 in the MET mice was significantly higher than that in the CON mice, though the levels of FSH showed no significant difference between the two groups (Figure 2). These results indicate that metformin could improve murine ovarian endocrine functions at the animals’ middle age.

**Table 2:** Effect of metformin on the estrous cycle of mice after 3 and 6 months of feeding.

	**Group**	**Regular estrous cycle (%, n/N)**	**Irregular cycle or cession (%, n/N)**
**0 month**	CON	100 (21/21)	0 (0/21)
	MET	100 (22/22)	0 (0/22)
**3 months**	CON	85.7 (18/21)	14.3 (3/21)
	MET	85 (17/20)	15 (3/20)
**6 months**	CON	38.5 (5/13)	61.5 (8/13)
	MET	78.9 (15/19)	21.1 (4/19)

### Metformin increases the ovarian reserve

**Figure 3:**
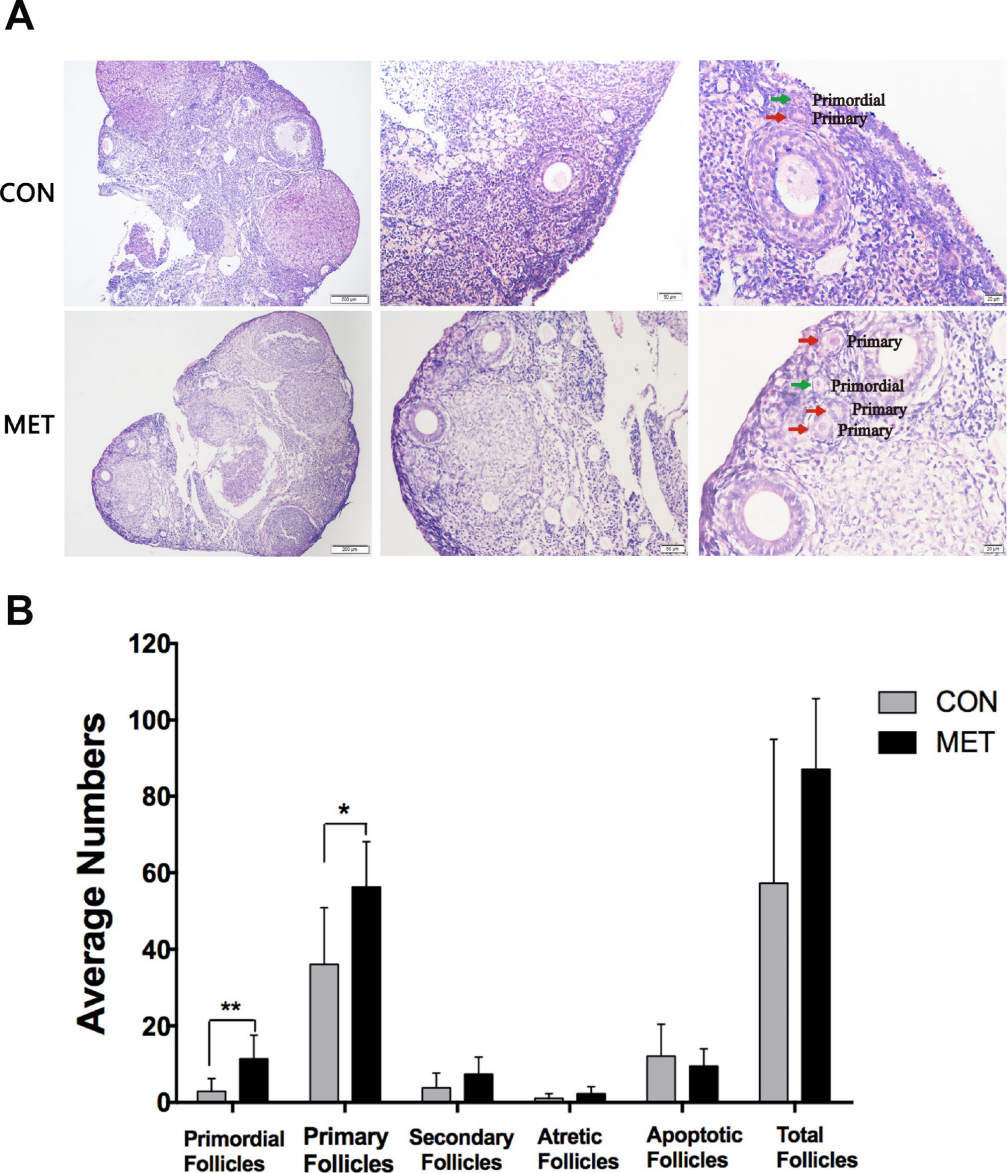
**Effect of MET feeding for 6 months on follicle numbers at different stages.** (**A**), The histological images of the ovaries in the CON and MET groups. The green and red arrows show primordial and primary follicles respectively. (**B**). The average number of follicles in each stage in the two groups. Data were presented as the mean± S.E.M. *indicates a significant difference from the CON group. *P<0.05, **P < 0.01.

Then, we tested the ovarian reserve on those mice by measuring the number of healthy ovarian follicles, as the proper ovarian function depends on sufficient ovarian reserve. We found that the MET mice exhibited more primordial and primary follicles than CON mice (P<0.05, indicating much higher ovarian reserve (Figure 3). This result demonstrates that metformin can increase ovarian reserve significantly.

### Metformin decreases oxidative damage and P16 in ovaries

To further analyze the molecular and cellular changes in murine ovaries after treatment with metformin, we measured the levels of the proteins 8-OhdG,NTY and 4-HNE as they are widely accepted as biomarkers of oxidative DNA, protein, and lipid damage respectively [22]. We found that the levels of both 8-OHdG and 4-HNE are decreased in MET murine ovaries compared to that in CON murine ovaries, though there is no difference in the NTY expression between the two groups.

**Figure 4:**
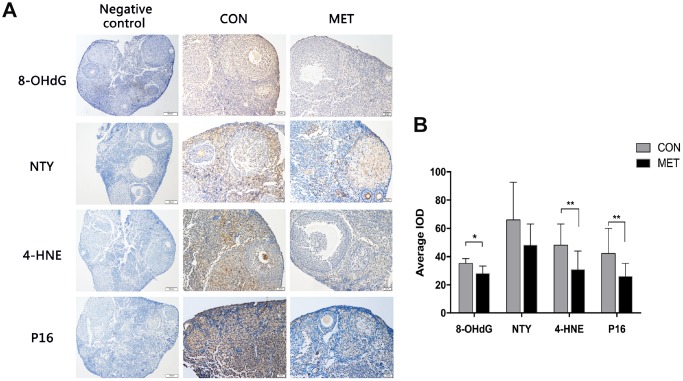
**Effects of MET on oxidative damage markers and the p16 level in both groups.** (**A**). The representative IHC images of p16 protein in the CON and MET mouse ovaries. (**B**). Average IOD of the markers in the two groups. Values are expressed as mean± S.E.M. *indicates a significant difference between the two group. *P<0.05, **P < 0.01.

We also tested the level of senescence-associated protein P16 by immunohistochemistry. The P16 expression was significantly lower in the MET murine ovaries than that in the CON group, indicating a relatively younger state of ovary after 6-month feeding with metformin (Figure 4). These results demonstrate that metformin can reduce oxidative damage and P16 levels in murine ovaries and thus maintain the organs in a relatively young age.

### Metformin induces SIRT1 level, but reduces p-rpS6 level

**Figure 5:**
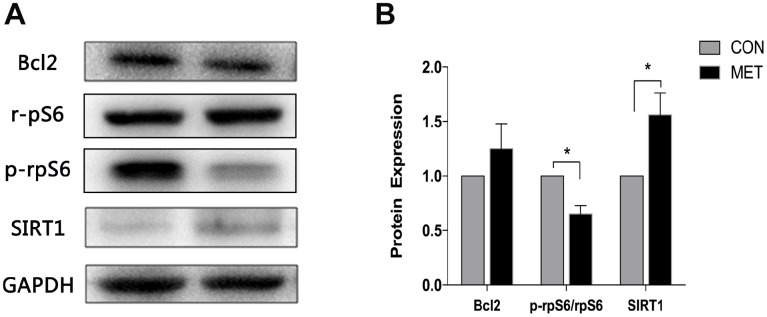
**Levels of SIRT1, Bcl2, p-rpS6 and rpS6 proteins in murine ovaries.** (**A**) Western blotting analyses of SIRT1, Bcl2, p-rpS6 and rpS6 with antibodies as indicated. (**B**) Quantitative analysis of data in A. Data were presented as the mean ± S.E.M. *compared with the NC group, *P < 0.05.

To investigate the potential underlying mechanisms for the aforementioned metformin’s role in protecting ovarian aging, we determined the levels of ovarian aging associated proteins, such as SIRT1, p-rpS6 and Bcl2 by immunoblotting analysis, as the expression of SIRT1 is closely associated with ovarian reserve [23]. As a result, we found that the SIRT1 level in MET mice was higher than that in CON mice. The p-rps6 protein plays a vital role in the activation and exhaustion of ovarian primordial follicle pool [24]. Our results showed that the level of p-rps6 protein was significantly decreased in the MET group, compared to the CON group (Figure 5). However, there was no difference in the expression of Bcl2 between the two groups of mice. Since previous studies showed that metformin can induce the expression of SIRT1 in other tissues [25], we therefore speculated that metformin might play an anti-aging role probably by inducing the expression of SIRT1 but reducing the level of p-rps6.

## DISCUSSION

Several studies have shown that metformin can improve the physical condition of mice [11]. However, the results from our experiments as described here showed no significant difference in the exercise ability and coordination ability between the CON and MET groups of mice, while the rate of learning in the MET mice was better than that in the CON mice as shown in the rotation experiment (Figure 1). Also, we found no significant difference in their body and organ weights between the two groups, although the incidence of abdominal masses in the CON group was higher than that in the MET group (data not shown), which was consistent with the reported studies [26, 27]. Generally consistent with previous studies [11], our results indicate that the moderate dosage of metformin admitted to mice for half a year is quite safe to the animals without any significant adverse effects on their physical activities and health.

Menstrual disorders are the mainly clinical manifestations of perimenopausal women, including irregular menstrual cycles, such as prolonged menstruation, increased or reduced menstruation. The vaginal epithelial cells of mice change periodically with the alteration of ovarian estrogen and progesterone levels as well. By observing the morphology and proportion of the vaginal cells, the estrous cycle can be estimated in mice. We found no significant difference in estrous cycles between the two groups of mice after feeding the animals with metformin for 3 months. However, after feeding the animals with metformin for 6 months, the proportion of regular estrous cycles in the MET group was significantly higher than that in the CON group, indicating that metformin could improve the endocrine function of middle-aged mice. This result is consistent with previous studies showing that metformin prolongs the life span and improve the estrous cycles of female mice [8, 28]. Consistently, the level of estrogen in the MET mice was significantly higher than that in the CON mice. Therefore, we believe that metformin can improve the endocrine function of an aging murine ovary.

Ovarian function decreases with age due to the diminished ovarian reserve. This was indeed the case in the CON mice with accelerated depletion of the follicle pool (Figure 3), which was consistent with natural aging mice. By contrast, metformin treatment for 6 months remarkably increased the amounts of the primordial and primary follicles in the MET group compared to those in the CON group, indicating that metformin could maintain the ovarian reserve. p16, a cell cycle dependent kinase inhibitor, prevents the cell cycle progression from G1 phase to S phase, and its expression increases with age [29]. Thus, it is considered to be an aging related indicator for the ovarian aging process as well [30]. We found that the level of p16 in the ovaries of the MET mice is lower than that in the CON mice, indicating that metformin could relieve the aging of the ovarian tissue in mice. Therefore, these results indicate that metformin could improve the ovarian reserve and delay the aging process of ovary.

Our previous study showed that the ovarian reserve and the expression of SIRT1 protein decline with age, and ovarian reserve is positively correlated with the increase of SIRT1 expression in mice [23]. Thus, the decrease of SIRT1 levels could be used as an indicator of ovarian aging. In our current study as described here, we found that the level of SIRT1 protein in the ovarian tissues of the MET mice is much higher than that in the CON mice. This result suggests that one of the molecules that might contribute to the maintenance of ovarian reserve and improvement of ovarian aging by metformin could be SIRT1. It has been shown that activated SIRT1 can inhibit oxidative damage and prevent production of ROS in the process of aging [22]. Indeed, our results showed that the expression of 8-OHdG and 4-HNE in the MET group was significantly lower than that in the control group. Together, these results suggest that metformin could increase ovarian reserve and delay ovarian aging by activating SIRT1, and reducing oxidative injury in middle-aged murine ovaries.

In ovarian tissues, mTOR can promote the growth and development of follicles by activating the downstream effector ribosomal protein S6 kinase (S6K1) and phosphorylated ribosomal protein S6 (p-rpS6), suggesting that the mTOR/PI3K/RPS6 pathway plays a role in ovarian reserve and follicle development [31]. However, our results showed that the level of p-rpS6 in the ovarian tissues of the MET mice is reduced, which may be due to the inhibition of mTOR, but we could not exclude other possibilities at this stage. Therefore, further dissection of the upstream signal molecules of the mTOR related pathway is necessary to determine whether metformin can reduce the activation of primordial follicles by inhibiting mTOR. Bcl2 is an anti-apoptotic protein, which can inhibit the apoptosis of granulosa cells in follicles and plays a crucial role in maintaining follicle growth [32]. Our WB analysis of its level in ovarian tissues showed no significant difference between the CON and MET groups. Thus, metformin may have no obvious anti-apoptosis effect on the murine ovaries.

In summary, our results as described here demonstrate that treatment of mice with a moderate concentration of metformin for half a year could increase ovarian reserve and improve ovarian function in middle aged mice, thus delaying ovarian aging by increasing the level of SIRT1 and reducing oxidative damage, although the underlying mechanisms need to be further elucidated. This study suggests that metformin could serve as a promising agent for improving women’s reproductive life, which could be a tempting project for future clinical research.

## MATERIALS AND METHODS

### Animal treatment

Female C57BL/6 mice of 27-week-old were purchased from Beijing HFK Bio-Technology Co., Ltd. (Beijing, PR China) and fed freely for one week to adapt to the environment. The vaginal smears of these mice were taken every morning for consecutive seven days. One week later, mice with regular estrous cycles and normal body weight (30.59±1.70g) were chosen and divided into two groups, the control group (CON, n=21) and the metformin-treated group (MET, n=22). Mice in the CON group were fed with food and water freely, and those in the MET group were fed with the chows supplemented with 100mg/kg metformin. Body weight was measured every week, and mice were sacrificed at the end of the experiment. Blood samples were centrifuged at 3000 rpm for 5 min, and serum was collected and stored at −80 °C until analysis. And heart, liver, spleen, kidney, uterus, ovary, and brain were separated and weighed. All procedures and protocols conducted on animals were approved by the ethics committee of Tongji Hospital, Tongji Medical College, Huazhong University of Science and Technology in the People's Republic of China.

### Open-field test

This test was conducted in a quiet environment. Mice were placed back to the operator in the center of the bottom of the wide-field reaction box, then the camera and timing were started. Five minutes later, the camera was stopped, and 75% alcohol was used to clean the box completely so as to wipe out the mice's residual urine, smell and hair. The experiment was repeated after alcohol completely volatilized. The average distance and velocity in the wide field experiment were recorded and analyzed.

### Rotarod test

Mice were placed on the rotating bar and rotated in the form of uniform acceleration (10-40r.p.m., 5min). When the mice dropped or time reached 5 minutes, the experiment was stopped and time is recorded. Each mouse was tested 10 times, with 5 minutes apart, and for 5 consecutive days. In the test, mice holding the stick and turning around, or automatic jumping were determined as unqualified situations and excluded from the results. The learning rate and latency to fall of the mice were calculated. Latency to fall was calculated as the average time to fall from the rotarod during the 10 trials for each mouse. Learning rate was calculated as follows (mean latency to fall _trials 9 and 10_)-(mean latency to fall _trials 1and 2_)/9 (number of intertrial intervals).

### Estrous cycle analysis

Vaginal smears of all mice were taken for consecutive seven days before the experiment began to exclude mice with irregular estrous cycles. During the experiment, vaginal smears were taken at about 9 am every day for consecutive 14 days after 3-month and 6-month feeding, respectively, to evaluate the estrous cycle condition. The vaginal secretion was taken with saline and then spread on the slides, followed by HE staining when it was dry. Then, we determined the stage of the estrous cycle by the cytology under microscope.

### Follicle counts

Six mice for both groups were used for follicle counts. Left ovary of each mice was preserved in 4% paraformaldehyde, then cut into pieces of 5um and put on the glass slides. Follicles in each stage were counted by two people separately. The detailed procedures were performed as previously described [33].

### Enzyme immunoassay for 17β-estradiol (E2) and progesterone (P)

Blood samples were collected when mice were sacrificed. Levels of serum E2 and P were measured by enzyme-linked immune sorbent assay (ELISA) according to the manufacturer's instructions (Cayman Chemical Company, Ann Arbor, USA).

### Western blot analysis

Western blot analysis was performed as previously described [34]. GAPDH was considered to be a loading control. Immunoreactive bands were visualized using alkaline phosphatase and BCIP/NBT staining. All blots were repeated three times.

### Immunohistochemical staining

Five representative sections from each of the ten selected ovaries per group were used for immunohistochemistry (IHC) to assess expression of P16, NTY, 4HNE, and 8-OdH. The ovaries were fixed in 4% paraformaldehyde for 24 h, then subsequently placed in 70% ethanol and bathed in paraffin. Ovaries were consecutively cut into 5μm, and every fifth section was transferred onto a slide. IHC analysis of ovarian tissues was performed using routine procedures as previously reported. Images were obtained using confocal microscopy (DM4000B; Leica, Germany).

### Statistical analysis

SPSS 17.0 software was used to conduct the statistical analyses. Group differences in body weight, length of estrous cycles, exhausted swimming time, hormone levels, follicle number counts and protein expression were investigated using Student’ s t-tests or non-parametric tests. Differences in the expression of P16 between the experimental and control groups were examined using the non-parametric Kruskal–Wallis test Values are represented as the mean ± SEM, and P< 0.05 was considered statistically significant.
